# Vesicular stomatitis virus-based vaccine targeting plasmodium blood-stage antigens elicits immune response and protects against malaria with protein booster strategy

**DOI:** 10.3389/fmicb.2022.1042414

**Published:** 2022-11-24

**Authors:** Yifan Sun, Xiaodan Shi, Feng Lu, Haitian Fu, Yi Yin, Jiahui Xu, Cheng Jin, Eun-taek Han, Xuan Huang, Yongquan Chen, Chunsheng Dong, Yang Cheng

**Affiliations:** ^1^Department of Laboratory Medicine, Affiliated Hospital of Jiangnan University, Wuxi, Jiangsu, China; ^2^Laboratory of Pathogen Infection and Immunity, Department of Public Health and Preventive Medicine, Wuxi School of Medicine, Jiangnan University, Wuxi, Jiangsu, China; ^3^Institute of Translational Medicine, Medical College, Yangzhou University, Yangzhou, China; ^4^Department of Nuclear Medicine, Affiliated Hospital of Jiangnan University, Wuxi, Jiangsu, China; ^5^Department of Hepatobiliary Surgery, Affiliated Hospital of Jiangnan University, Wuxi, Jiangsu, China; ^6^Department of Medical Environmental Biology and Tropical Medicine, School of Medicine, Kangwon National University, Chuncheon, Gangwon-do, South Korea; ^7^Wuxi School of Medicine, Jiangnan University, Wuxi, Jiangsu, China; ^8^Jiangsu Key Laboratory of Infection and Immunity, Institutes of Biology and Medical Sciences, Soochow University, Suzhou, Jiangsu, China

**Keywords:** *Plasmodium*, vaccine, vesicular stomatitis virus, AMA1, RH5, RON2

## Abstract

Merozoite invasion of the erythrocytes in humans is a key step in the pathogenesis of malaria. The proteins involved in the merozoite invasion could be potential targets for the development of malaria vaccines. Novel viral-vector-based malaria vaccine regimens developed are currently under clinical trials. Vesicular stomatitis virus (VSV) is a single-stranded negative-strand RNA virus widely used as a vector for virus or cancer vaccines. Whether the VSV-based malarial vaccine is more effective than conventional vaccines based on proteins involved in parasitic invasion is still unclear. In this study, we have used the reverse genetics system to construct recombinant VSVs (rVSVs) expressing apical membrane protein 1 (AMA1), rhoptry neck protein 2 (RON2), and reticulocyte-binding protein homolog 5 (RH5), which are required for *Plasmodium falciparum* invasion. Our results showed that VSV-based viral vaccines significantly increased *Plasmodium*-specific IgG levels and lymphocyte proliferation. Also, VSV-PyAMA1 and VSV-PyRON2sp prime-boost regimens could significantly increase the levels of IL-2 and IFN-γ-producing by CD4^+^ and CD8^+^ T cells and suppress invasion *in vitro*. The rVSV prime-protein boost regimen significantly increase *Plasmodium* antigen-specific IgG levels in the serum of mice compared to the homologous rVSV prime-boost. Furthermore, the protective efficacy of rVSV prime protein boost immunization in the mice challenged with *P. yoelii* 17XL was better compared to traditional antigen immunization. Together, our results show that VSV vector is a novel strategy for malarial vaccine development and preventing the parasitic diseases.

## Introduction

In 2020, approximately 241 million new cases of malaria and 627,000 malaria-related deaths were reported worldwide (WHO, 2021). *Plasmodium falciparum* (*P. falciparum*) is a highly prevalent malarial parasite in sub-Saharan Africa and is a major cause of malaria-related death (WHO, 2021). In the past few decades, continuous efforts have been made to substantially reduce the incidences and deaths associated with malaria by using artemisinin-based combination therapy and long-lasting insecticide-treated nets (WHO, 2021). However, the primary cause for the failure to completely eradicate malaria is the *Plasmodium* parasite’s resistance to frontline drugs and tolerance of mosquitoes to insecticides (Haldar et al., 2018; Minetti et al., 2020). Hence, there is a need to develop vaccines to prevent the occurrence and spread of malaria (Laurens, 2018; Stanisic and McCall, 2021). RTS,S/AS01 vaccine targets the circumsporozoite antigen of *P. falciparum* and is currently in pilot implementation in countries endemic to malaria since 2019. However, additional studies are required to evaluate the overall efficacy and safety profile of this vaccine (Laurens, 2020; Chatterjee and Cockburn, 2021). Further, the development and optimization of malaria vaccine strategies are required.

The micronemes, rhoptries, and dense granules are apical organelles of the *Plasmodium* parasite, which play key roles in the erythrocyte invasion. The apical organelle proteins are considered potential candidates for anti-malarial vaccines (Salinas et al., 2019). *P. falciparum* reticulocyte binding-like protein RH5 (PfRH5) is an apical organelle protein and a member of the erythrocyte ligands superfamily, which is essential for erythrocyte invasion (Bustamante et al., 2013; Douglas et al., 2014; Patarroyo et al., 2020). In cultured parasite lines, PfRH5 is typically processed by removing long disordered regions to generate a ~ 45 kDa fragment called PfRH5ΔNL. The fragment PfRH5ΔNL encompasses 140–526 aa residues but lacks 248–296 aa residues, which bind to basigin and play an inhibitory role in parasite invasion (Wright et al., 2014; Payne et al., 2017; Ragotte et al., 2020; Moore et al., 2021). Therefore, PfRH5 could be a potential target for developing a vaccine against blood-stage *Plasmodium* infection (Ragotte et al., 2020). Furthermore, immunization with adenovirus/poxvirus vector-based protein-in-adjuvant RH5 has been observed to induce immune responses against *P. falciparum* (Douglas et al., 2015).

AMA1 is a micronemal protein of apicomplexan parasites. As a structurally conserved type I integral membrane protein, PfAMA1 is necessary for the invasion of erythrocytes (Tyler et al., 2011). The anti-PfAMA1 antibodies, which primarily recognize domain I (DI) and domain II (DII), have been observed to induces high levels of growth-inhibitory antibodies (Lalitha et al., 2004). In addition, *Plasmodium* rhoptry neck protein 2 (RON2) is a receptor for AMA1, and the AMA1-RON2 complex serves as a strong anchoring point to inhibit host erythrocyte invasion (Srinivasan et al., 2011; Patarroyo et al., 2020). *In vivo* studies have shown that mice immunized with an AMA1-RON2 peptide complex could provide complete protection against the lethal challenge of *Plasmodium yoelii* (*P. yoelii*) compared to immunization with AMA1 alone (Srinivasan et al., 2014). Further, antibodies generated in monkeys immunized with the AMA1-RON2L complex demonstrated enhanced neutralizing potency (Srinivasan et al., 2017). A previous study has shown that antibodies against the AMA1-RON2L/RH5 combination could consistently generate an additive growth-inhibitory effect against *P. falciparum,* as demonstrated using a growth inhibition assay (GIA) (Azasi et al., 2020). Therefore, *Plasmodium* RH5 and AMA1-RON2 combinations serve as potential antigen targets for the development of novel malarial vaccines.

The vesicular stomatitis virus (VSV) belongs to the *Rhabdoviridae* family and is an enveloped, nonsegmented, single-negative-strand RNA virus. In this study, we have used a reverse genetics system to construct recombinant VSVs (rVSVs) expressing a VSV nucleocapsid protein and two polymerase subunits to maintain the replicative ability of the virus (Lawson et al., 1995). In addition, the rVSVs expressing foreign antigens without altering its growth characteristics, which could benefit for vaccine development (Lawson et al., 1995). VSV have been developed as a vaccine vector for multiple pathogens, including bacteria, DNA, and RNA viruses. This could aid in inducing robust cellular and humoral immune responses and confer protection against challenges in animal models (Humphreys and Sebastian, 2018; Fathi et al., 2019). The Food and Drug Administration has approved the rVSVΔG-ZEBOV-GP vaccine for the prevention of Ebola virus disease (Choi et al., 2021). This indicates that VSV could serve as a robust vector backbone for vaccines and can be used against infectious diseases (Fathi et al., 2019). Recently, various studies have used different VSV-based strategies to develop vaccines against SARS-CoV-2 and the protection effect mainly due to affecting the cell entry and inducing neutralizing antibodies (Case et al., 2020; Yahalom-Ronen et al., 2020). The VSV vector has a simple structure as well as genetic makeup and can induce high virus titer. It is mildly pathogenic, has a short immunization period, and has a good safety profile, thus making rVSV a desirable vector for vaccine development (Li et al., 2007; Fathi et al., 2019). However, VSV as a vector for malaria vaccine development has not been explored.

In the current study, we first constructed a novel malaria vaccine using blood-stage antigens as immunogens and VSV as the vector. Our results showed that the VSV-PfRH5ΔNL or VSV-PfAMA1_345_ + VSV-PfRON2sp immunization strategy in mice induced specific antibodies and polyfunctional T cell responses in mice. These candidate vaccines effectively suppressed the invasion of *P. falciparum in vitro*. Furthermore, our data showed that rVSVs (VSV-PyAMA1_343_ + VSV-PyRON2sp) prime and protein boost strategy stimulated T cells to secrete high levels of IFN-γ and IL-2 compared to protein-only vaccination. However, no significant differences in parasitemia and survival rate of mice were observed between the two vaccination strategies. Interestingly, both vaccines could protect against *P. yoelii* challenge in mice. Our results showed that rVSVs expressing *Plasmodium* blood-stage antigens as candidates for malaria vaccines and extended the potential application of VSV vector vaccine.

## Materials and methods

### Cell culture

Vero cells are kidney epithelial cells derived from *Cercopithecus aethiops*, and BSR-T7 cells are kidney cells stably expressing T7 polymerase derived from baby hamsters (Buchholz et al., 1999). Vero cells and BSR-T7 cells were cultured in Dulbecco’s Modified Eagle’s medium (DMEM) (Hyclone, UT, USA) containing 10% fetal bovine serum (Gibco, NY, USA) and supplemented with 1% penicillin–streptomycin solution (Gibco). All cells were maintained at 5% CO_2_ in a humidified incubator at 37°C.

### Generation of rVSVs

**Figure 1:**
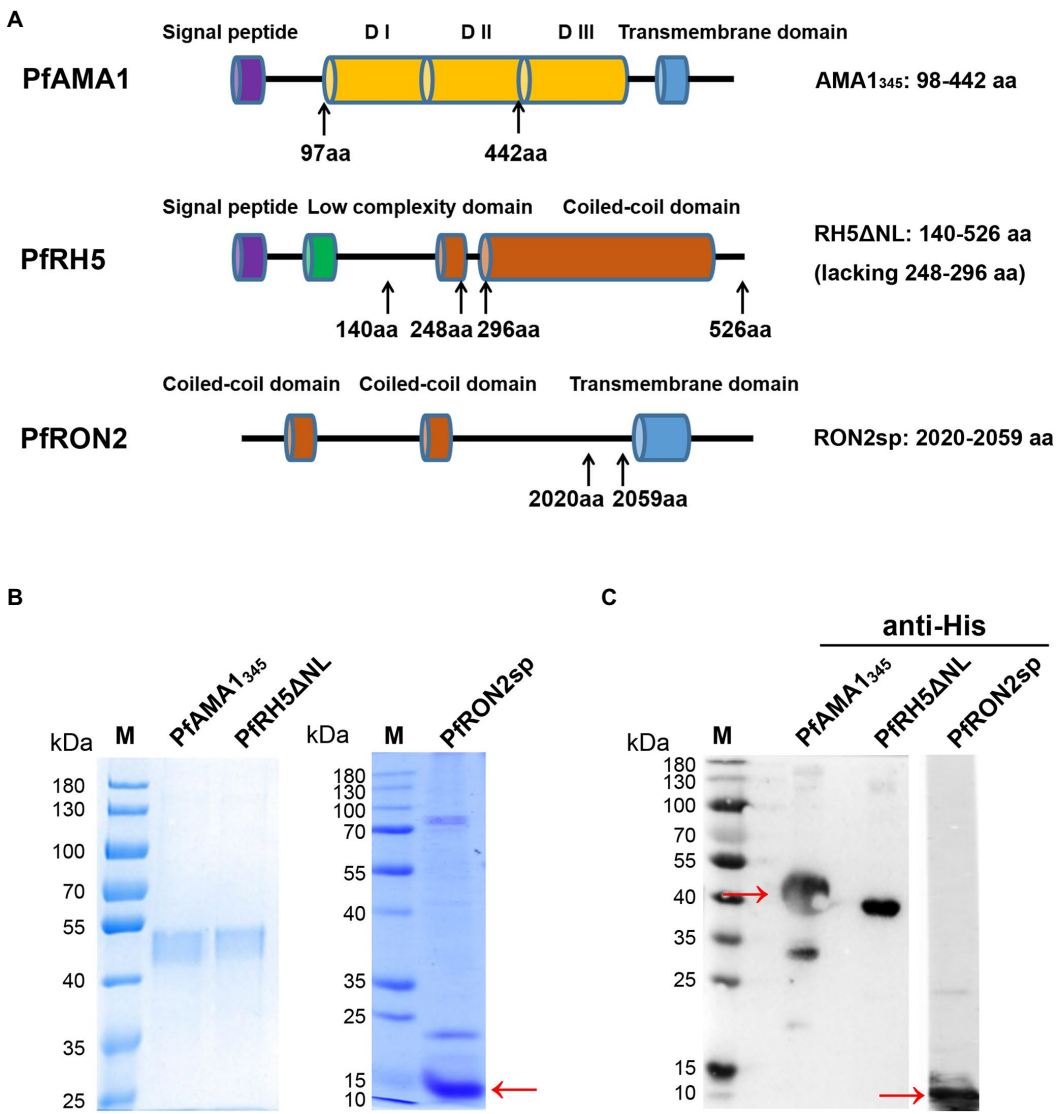
Construction, expression, and purification of recombinant PfAMA1_345_ and PfRH5ΔNL proteins, as well as synthesis and identification of PfRON2sp. **(A)** Schematic representation of PfAMA1, PfRH5, and PfRON2. The signal peptide is shown in purple, domain I/ domain II/ domain III (D I/D II/D III) in orange, the transmembrane domain in blue, the low complexity domain in green, and the coiled-coil domain in red. The genes encoding PfAMA1_345_ (98-442aa) and PfRH5ΔNL (140-526aa, lacking 248-296aa) were cloned for expression and purification. PfRON2sp (2020-2059aa) peptide was synthesized. The amino acid residue of PfAMA1_345_, PfRH5ΔNL, and PfRON2sp were shown in the right panel. **(B)** Expression and purification of PfAMA1_345_ and PfRH5ΔNL proteins. Codon-optimized PfAMA1_345_ and PfRH5ΔNL genes were cloned in the pET28a (+) vector, transformed in *E. coli*, and purified using Ni-sepharose beads. The purified PfAMA1_345_, PfRH5ΔNL, and PfRON2sp peptides were separated by SDS-PAGE and stained using Coomassie brilliant blue **(B)** and immunoblotting with anti-His antibody for PfAMA1_345_, PfRH5ΔNL, and anti-RON2 for PfRON2sp **(C)**.

Figure 1A shows the rVSVs containing AMA1_345_ (PF3D7_1133400, Domain I, and Domain II, residues 98–442 aa), RH5ΔNL (PF3D7_0424100, residues 140–526 aa, but lacking residues 248–296 aa), and RON2sp (PF3D7_1452000, C-terminal region residues 2020–2059 aa) from *P. falciparum* 3D7. The second construct was rVSVs containing AMA1_343_ (PYYM_0916000, Domain I, and Domain II, residues 43–385 aa) and RON2sp (PYYM_1316500, residues 1839–1877 aa) from *P. yoelii* 17XL (Py17XL). The codon-optimized antigen-encoding sequences, encompassed the bases expressing Flag-tag, were synthesized with the base by Talen Biotech (Shanghai, China). The restriction enzymes *Xho* I and *Nhe* I were used to insert the sequences between the G and L genes of the VSV expression vector pXN2. The recombinant VSVs were recovered using a reverse genetic system (Publicover et al., 2004). Briefly, 3 × 10^6^ Vero cells were seeded in 10 cm dishes and allowed to adhere overnight. Vero cells were infected with the vaccinia virus expressing the T7 RNA polymerase (Fuerst et al., 1986) at a multiplicity of infection (MOI) of 2.5 in serum-free DMEM medium and incubated for 2 h. The cells were transfected using the Lipofectamine™ LTX Reagent (Invitrogen, CA, USA) with 10 μg of recombinant pXN2 and 4 μg of other plasmids encoding VSV nucleocapsid (N), 5 μg phosphoprotein (P), and 2 μg large polymerase subunit (L) according to manufacturer’s instruction. BSR-T7 cells supernatant was collected after two days of transfection and filtered using a 0.22 μm filter (Millipore, MA, USA) to remove the vaccinia virus. This supernatant was used to infect new BSR-T7 cells. BSR-T7 cytopathy was observed after two days of infection. The rVSVs released into the supernatant were collected and stored at −80°C. The rVSVs were replicated in Vero cells, and the virus titer was determined by 50% tissue culture infective dose(TCID50). The rVSVs titers obtained were within the range of 10^7^ ~ 10^9^ PFU. The recombinant VSV-green fluorescent protein (VSV-GFP) was used as a control and cloned as described above. The packaging plasmids pXN2-GFP, pP, pL, and pN were gifted by Prof. John Rose (Yale University).

### Expression, purification of proteins, and immunization

The gene fragments AMA1_345_, RON2sp, and RHΔNL from *P. falciparum* 3D7 and AMA1_343_ and RON2sp from Py17XL (same as VSV constructs) were synthesized by Talen Biotech (Shanghai, China). These gene fragments were cloned into the pET28a (+) vector after codon optimization. The cloned vector was sequenced to determine if the sequence assembly was accurate by DNA sequencing. The plasmids were transformed into *Escherichia coli* BL-21 cells to produce recombinant proteins with the His-Tag. The proteins were induced using 0.1 mM IPTG at 37°C and purified using Ni-sepharose beads (YouLong Biotech, Shanghai, China). The endotoxin from the recombinant proteins was removed prior to subsequent use. The purity of the proteins was >80% which was determined by YouLong Biotech. The PfRON2sp polypeptide was synthesized by YouLong Biotech (Shanghai, China), and the purity of the peptide was analyzed by high-performance liquid chromatography (HPLC). All antisera were raised in mice using standard protocol by YouLong Biotech (Shanghai, China).

### SDS-PAGE and Western blotting

The purified recombinant proteins were separated and visualized using 10% sodium dodecyl sulfate-polyacrylamide gel electrophoresis (SDS-PAGE) stained with Coomassie brilliant blue. The proteins were separated on 10% SDS-PAGE and transferred to polyvinylidene difluoride (PVDF) membranes (Amersham Biosciences, NJ, USA). The PVDF membranes were blocked with 5% skim milk for 1 h at room temperature, followed by incubation with primary anti-His antibodies (Abcam, Cambridge, UK), or antisera at 4°C overnight. The membranes were washed with tris-buffered saline containing 0.1% Tween 20 (TBST) and incubated with 1:5000 dilution horseradish peroxidase-conjugated secondary antibody (Southern Biotech, UAB, USA). The signal was detected with a chemiluminescence reagent (Thermo, MA, USA) using the ImageQuant LAS4000 system (GE Healthcare, Piscataway, NJ, USA). BSR-T7 cells were transfected with recombinant VSVs (MOI = 10) for 12 h. The cells were lysed using RIPA buffer containing 1 mM PMSF (Sigma, MO, USA) for 30 min at 4°C. The lysate was boiled for 5 min, and the proteins were separated on 10% SDS-PAGE. Western blotting was performed using primary anti-flag antibodies (Abcam, Cambridge, UK) or anti-PfRON2sp antiserum.

### Indirect immunofluorescence assay

BSR-T7 cells were infected with rVSVs at MOI = 10. After 12 h of infection, the cells were collected, and 1 × 10^5^ cells were seeded in a micro-well plate. The cells were fixed with ice-cold acetone for 5 min and blocked using 5% skim milk in phosphate buffer saline (PBS) at 4°C overnight. The cells were incubated with antisera from recombinant protein (anti-PfRH5 and anti-PfAMA1) or peptide (anti-PfRON2)-immunized mouse or rabbit at 1:200 dilution in PBS for 1 h at 37°C. The cells were incubated with Alexa Fluor 546-conjugated goat anti-mouse IgG or Alexa Fluor 488-conjugated goat anti-rabbit IgG secondary antibodies (Invitrogen) for 1 h at 37°C. The nuclei were stained with 4′, 6-diamidino-2-phenylindole (DAPI; Invitrogen) at 37°C for 30 min and mounted using a ProLong Gold antifade reagent (Invitrogen). The cells were visualized under oil immersion using a confocal laser scanning FV200 microscope (Olympus, Tokyo, Japan) equipped with ×20 dry and ×60 oil objectives. The images were captured with FV10-ASW 3.0 viewer software and prepared for publication with Adobe Photoshop CS5 (Adobe Systems, CA, USA). For immunofluorescence on *Pf* parasite lysate, the *Pf* 3D7 strain was purified using 63% Percoll. The cells were blocked with PBS containing 5% nonfat milk. The cells were incubated with rabbit anti-sera of AMA1, RH5, and RON2, which were used as primary antibodies, followed by incubation with Alexa Flour 546-conjugated goat anti-rabbit IgG (Invitrogen). The cells were counterstained with DAPI and mounted using ProLong Gold antifade reagent. The parasites were visualized under oil immersion using a confocal laser scanning FV200 microscope (Olympus).

### Animal vaccination

6–8 weeks old male BALB/c mice were obtained from Shanghai SLAC Laboratory Animal Co. Ltd. (Shanghai, China). The mice were maintained under “specific pathogen-free” conditions per the guidelines established by Jiangnan University Institutional Animal Care and Use Committee (Wuxi, China). To determine the immune response induced by rVSVs expressing *P. falciparum* antigens, BALB/c mice were vaccinated *via* intranasal routes with 10^6^ plaque-forming units (PFU) VSV-PfRH5ΔNL or 5 × 10^5^ PFU VSV-PfAMA1_345_ and VSV-PfRON2sp in 1:1 ratio (*n* = 5 mice/group). 25 μl of the vector was used to immunize the mice on day 0, and a booster dose was administered on day 14. The mice vaccinated with VSV-GFP and PBS were used as controls. For immunization with purified proteins, 50 μg PfRH5ΔNL proteins/mice or 25 μg PfAMA1_345_/mice +25 μg PfRON2sp proteins or peptides dissolved in 100 μl PBS with an equal volume of complete Freund’s adjuvant were injected intraperitoneally in mice. Freund’s incomplete adjuvant was administered on days 14 and 28 to boost immunity. The injections were administered thrice at an interval of 2 weeks.

To investigate the protective efficacy of the vaccines against *P.yoelii* infection, 6–8 weeks old BALB/c mice were divided into five groups and vaccinated with rVSVs through the intranasal route. The proteins were injected intraperitoneally. Group 1 and 2 mice were vaccinated with VSV-GFP and PBS, respectively, and served as controls. Group 3 mice (rVSVs boosting with rVSVs, rVSVs-rVSVs) were primarily immunized with 25 μl of 10^6^ PFU VSV-PyAMA1_343_ + VSV-PyRON2sp in 1:1 ratio (5 × 10^5^ PFU each, n = 5 mice/group) and booster dose was administered day 14. Group 4 mice (rVSVs boosting with double protein immunizations, rVSVs-P–P) were first immunized with 25 μl of 10^6^ PFU VSV-PyAMA1_343_ + VSV-PyRON2sp on day 0. The booster dose consisted of 50 μg PyAMA1_343_ + PyRON2sp proteins (25 μg each) dissolved in 100 μl PBS with an equal volume of incomplete Freund’s adjuvant administered on days 14 and 28. Group 5 mice (Triple protein immunization, P-P-P) were immunized with a total of 50 μg PyAMA1_343_ + PyRON2sp proteins (25 μg each) dissolved in 100 μl PBS with an equal volume of complete Freund’s adjuvant on day 0. The booster dose comprised the same proteins with incomplete Freund’s adjuvant and was administered on days 14 and 28. Group 4 and 5 mice were vaccinated with VSV-GFP and PBS, respectively, and served as controls. All animal experiments were approved by the Animal Ethics Committee of Jiangnan University [JN. No. 20180615t0900930 (100)].

### Enzyme-linked immunosorbent assay

To measure antigen-specific IgG responses, the serum was collected from the immunized mice from a tail vein on days 0, 7, 21, and 35 after first immunization with *P. falciparum* antigens combinations and day 35 after first immunization with *P. yoelii* antigens combinations. First, we coated with PfAMA1 and PfRON2 protein (peptides) for VSV-PfAMA1_345_ + VSV-PfRON2sp and PfAMA1_345_ + PfRON2sp immunization and coated with PfRH5ΔNL protein for VSV-PfRH5ΔNL and PfRH5ΔNL immunization. Similarly, we coated with PyAMA1_343_ and PyRON2sp protein (peptides) for VSV-PyAMA1_343_ + VSV-PyRON2sp and PyAMA1_343_ + PyRON2sp immunization. 96-well polystyrene microplates (Corning, NY, USA) were coated with the corresponding antigens from *P. falciparum* 3D7 [5 μg/ml of PfRH5ΔNL, PfAMA1_345_ + PfRON2sp proteins (peptides)], and *P. yoelii* [5 μg/ml of PyAMA1_343_ + PyRON2sp proteins (peptides)] with 100 μl/well coating buffer (Na_2_CO_3_, 50 mM, pH9.6) overnight at 4°C. The antigenic sites were blocked with 5% bovine serum albumin in PBS for 2 h at 37°C. 1:40 diluted serum was added to the antigen-coated plates and incubated for 2 h at 37°C, followed by incubation with HRP-conjugated goat anti-mouse IgG secondary antibody (diluted at 1:3000) for 1.5 h at 37°C. The signals were detected using a tetramethylbenzidine kit (Sigma, MO, USA) at room temperature, and the reactions were terminated using 2 M H_2_SO_4_. The optical density of the solution was measured at 450 nm using a Multiskan FC microplate reader (Thermo Fisher Scientific, MA, USA). One-way analysis of variance (AVONA) was used to test the differences.

### MTT assay

BALB/c mice from all groups were euthanized on day 35 post-first immunization, and the splenic lymphocytes were harvested. 100 μl of 5 × 10^5^ cells/well splenic lymphocytes were seeded in 96-well plates. The cells were then stimulated with 5 μg/ml PfRH5ΔNL or PfAMA1_345_ + PfRON2sp proteins for 72 h. Next, 50 μg thiazolyl blue tetrazolium bromide (MTT, Beyotime, Shanghai, China) was added to each well and incubated for 4 h. To terminate the reaction100 μl, dimethyl sulfoxide was added to each well and incubated for 10 min. The absorbance was measured at 570 nm using a Multiskan FC microplate reader (Thermo Fisher Scientific). One-way AVONA was used to test the differences.

### Flow cytometry

BALB/c mice from all groups were sacrificed on day 35 post-first immunization. The splenocytes were isolated, and the red blood cells were harvested using ACK lysis for 3 min at room temperature. The cells were washed with PBS and centrifuged at 1500 rpm for 5 min. Next, the cell pellet was resuspended in RPMI 1640 complete medium, and 5 × 10^5^ cells/100 μL/well were seeded in 96-well plates. The cells were stimulated with 5 μg/ml PfRH5ΔNL and PfAMA1_345_ + PfRON2sp proteins (peptides) for 24 h, followed by treatment with 50 ng/ml phorbol 12-myristate 13-acetate (PMA; Sigma), 1 μg/ml ionomycin (Sigma), and 1 μg/ml bafilomycin A (Sigma) for 6 h. Next, the cells were incubated with allophycocyanin-conjugated-anti-mouse CD4 and fluorescein isothiocyanate-conjugated-anti-mouse CD8 (Biolegend, CA, USA) for surface staining. The cells were fixed, permeabilized (BD Biosciences, NJ, USA), and stained with phycoerythrin (PE)-conjugated anti-IFN-γ and PE/Cy7-conjugated anti-IL-2 antibodies (Biolegend) to detect interferon (IFN)-γ and interleukin (IL)-2. The cells were sorted using a FASCanto II flow cytometer (BD Biosciences). The splenocytes of mice immunized with antigens derived from *P.yoelii* were isolated on day 10 post-immunization and stimulated with 5 μg/ml PyAMA1_343_ + PyRON2sp proteins. The rest of the protocol is the same as those described above for flow cytometry. One-way AVONA was used to test the differences in cytokine levels in mice immunized with antigens derived from *Pf*. The student’s t-test was used to assess the differences in cytokine levels in mice immunized with antigens derived from *Py*.

### Growth inhibition assay

*Plasmodium falciparum* 3D7 parasites were cultured in human O^+^ erythrocytes at 5% hematocrit. Next, the synchronized parasites were collected on day 35 after the first immunization for 24 h at 37°C and incubated with antisera at 1:100, 1:1000, or 1:2000 dilutions. The inhibition assays were carried out in 96-well plates for all strains and antibody concentrations, and the experiment was carried out in triplicates. The cultures were fixed, and parasitized erythrocytes in at least 30 high-power fields were counted using a microscope. A 5% sorbitol solution was used to synchronize the parasites into the ring stage and incubated in the presence of antiserum at the same concentrations indicated above for 24 h at 37°C. The cells were fixed with 0.05% glutaraldehyde and stained using SYBR Green I nucleic acid gel stain (Invitrogen). 1 × 10^6^ cells were used to perform inhibition assays using flow cytometry. The invasion inhibition efficiency was calculated as described previously (Lu et al., 2022). One-way AVONA was used to test the differences.

### *Plasmodium yoelii* 17XL challenge

The mice were infected with Py17XL by administering 5 × 10^5^ parasitized erythrocytes (perythrocytes) on day 35 after the initial immunization. Blood smear microscopy was used to determine parasitemia every day post-infection. Briefly, a drop of blood was smeared on a glass slide, dried, and stained using a Wright’s-Giemsa staining kit (JianCheng, Nanjing, China). The slides were observed under an oil microscope. Once all the PBS-treated mice had died, the surviving mice from the other groups were sacrificed on day 10 post-infection. The splenocytes from mice were harvested to analyze the cytokine secretion levels. The mice were observed for 10 days to determine the survival rates. According to the Animal ethics guidelines, humane endpoints were considered in all the *in vivo* experiments. Two-way AVONA was used to test the differences in parasitemia. Log-ranks (Mantel-Cox) tests were used to study the survival rates in mice.

### Statistical analysis

GraphPad Prism software (version 5.0) was used to analyze data and create graphs. One-way or two-way ANOVA was used to perform statistical analysis. Log-ranks (Mantel-Cox) tests were used to study the survival rates in mice. *p* < 0.05 was considered statistically significant.

## Results

### Expression and purification of recombinant proteins using the *Escherichia coli*

A schematic diagram of PfAMA1_345_ (98–442 aa), PfRH5ΔNL (140–526 aa, lacking 248–296aa), and PfRON2sp (2020–2059 aa) is shown in Figure 1A. PfAMA1_345_ and PfRH5ΔNL proteins were expressed using the *E. coli* and purified. PfAMA1_345_ and PfRH5ΔNL proteins were separated using SDS-PAGE and stained with Coomassie brilliant blue (Figure 1B). The protein expression was determined using western blotting (Figure 1C). In addition, PfRON2sp was synthesized and identified by SDS-PAGE, western blotting (Figures 1B,C), and HPLC (Supplementary Figure S1).

### Generation of recombinant VSV-PfAMA1_345_, VSV-PfRH5ΔNL, and VSV-PfRON2sp

**Figure 2:**
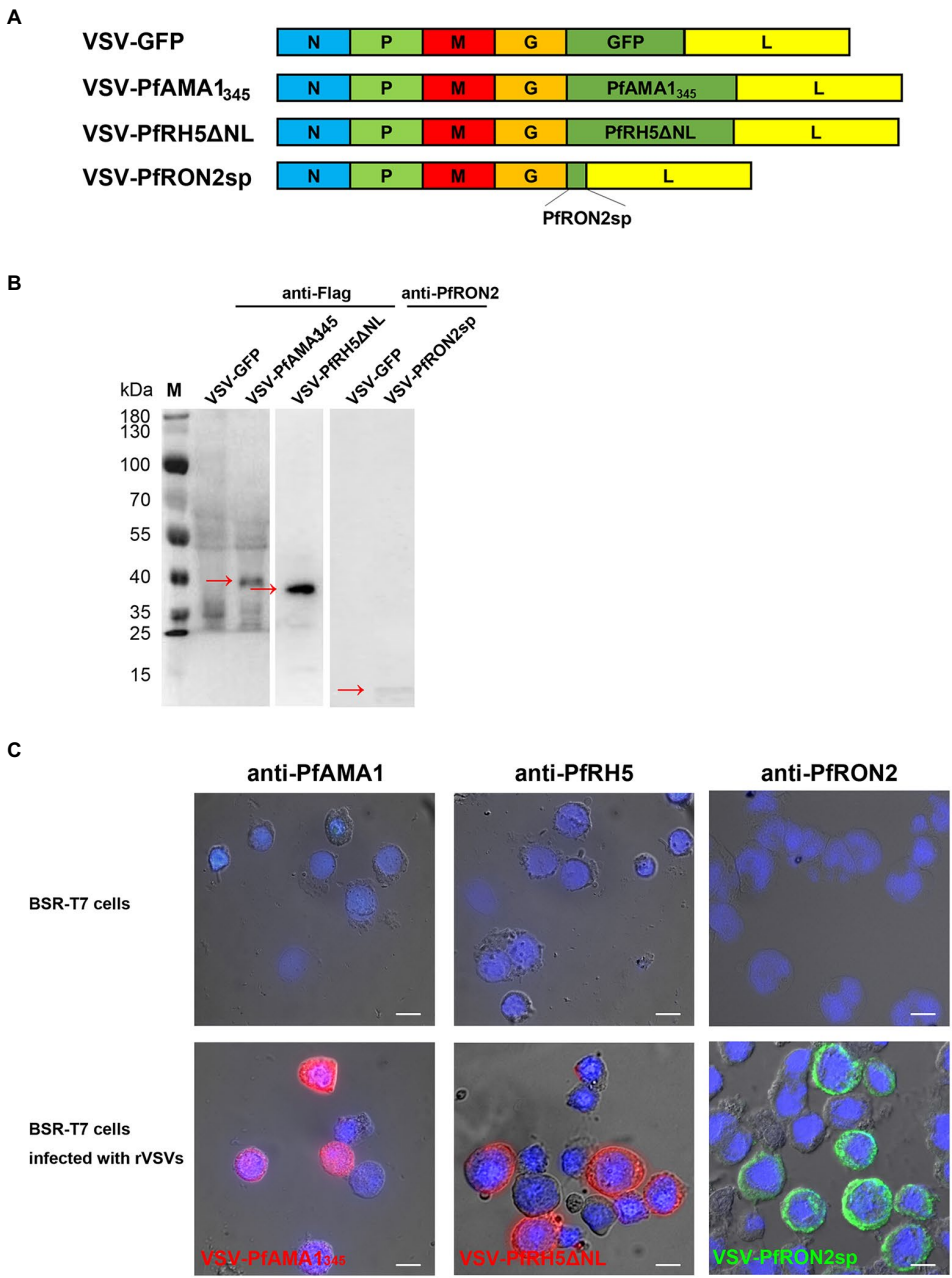
Construction and characterization of recombinant VSV-PfAMA1_345_, VSV-PfRH5ΔNL, and VSV-PfRON2sp. **(A)** Construction of VSV-PfAMA1_345_, VSV-PfRH5ΔNL, VSV-PfRON2sp, and VSV-GFP. Gene encoding *Plasmodium falciparum* antigen PfAMA1_345_, PfRH5ΔNL, and PfRON2sp were cloned into VSV vector pXN2 between the G and L genes. The recombinant VSV pseudotype was cloned into pXN2-GFP, recombinant pXN2, and other plasmids encoding VSV nucleocapsid (N), phosphoprotein (P), and large polymerase subunit (L) to reconstruct the VSV genome. **(B)** BSR-T7 cells infected with rVSVs were harvested and analyzed 12 h post-infection by western blotting using anti-Flag antibody for VSV-PfAMA1_345_ and VSV-PfRH5ΔNL and anti-PfRON2 antisera for VSV-PfRON2sp. BSR-T7 cells infected with VSV-GFP were used as the control. **(C)** Indirect immunofluorescence was performed to study PfAMA1_345_, PfRON2sp, and PfRH5ΔNL expression in BSR-T7 cells infected with recombinant VSVs 12 h post-infection using anti-PfAMA1, PfRH5, or PfRON2sp antisera obtained from antigen-immunized mice. Non-transfected BSR-T7 cells were used as the control. The bar represents 10 mm.

The codon-optimized antigen-encoding sequences were cloned into the pXN2 vector at the G–L junction in the VSV genome using *Xho* I and *Nhe* I restriction enzymes (Figure 2A). The rVSVs were packaged using the reverse genetic system in BSR-T7 cells. The cytopathic effect caused by rVSVs indicated that rVSVs were successfully packaged in BSR-T7 cells. VSV-GFP served as a control (Supplementary Figure S2A). The expression of PfAMA1_345_ and PfRH5ΔNL proteins with Flag-tag was confirmed using western blot (Figure 2B). In addition, the expression of PfRON2sp was determined using anti-PfRON2sp antiserum obtained from PfRON2sp peptide-immunized mice (Figure 2B). Immunofluorescence results demonstrated cytoplasmic localization of PfAMA1_345_, PfRH5ΔNL, and PfRON2sp proteins in rVSV-infected BSR-T7 cells (Figure 2C). Together, these results demonstrate that recombinant viruses like VSV-PfAMA1_345_, VSV-PfRH5ΔNL, and VSV-PfRON2sp were successfully cloned and packaged.

### rVSVs containing PfAMA1_345_, PfRON2sp, and PfRH5ΔNL induce specific humoral and cellular immune responses

**Figure 3:**
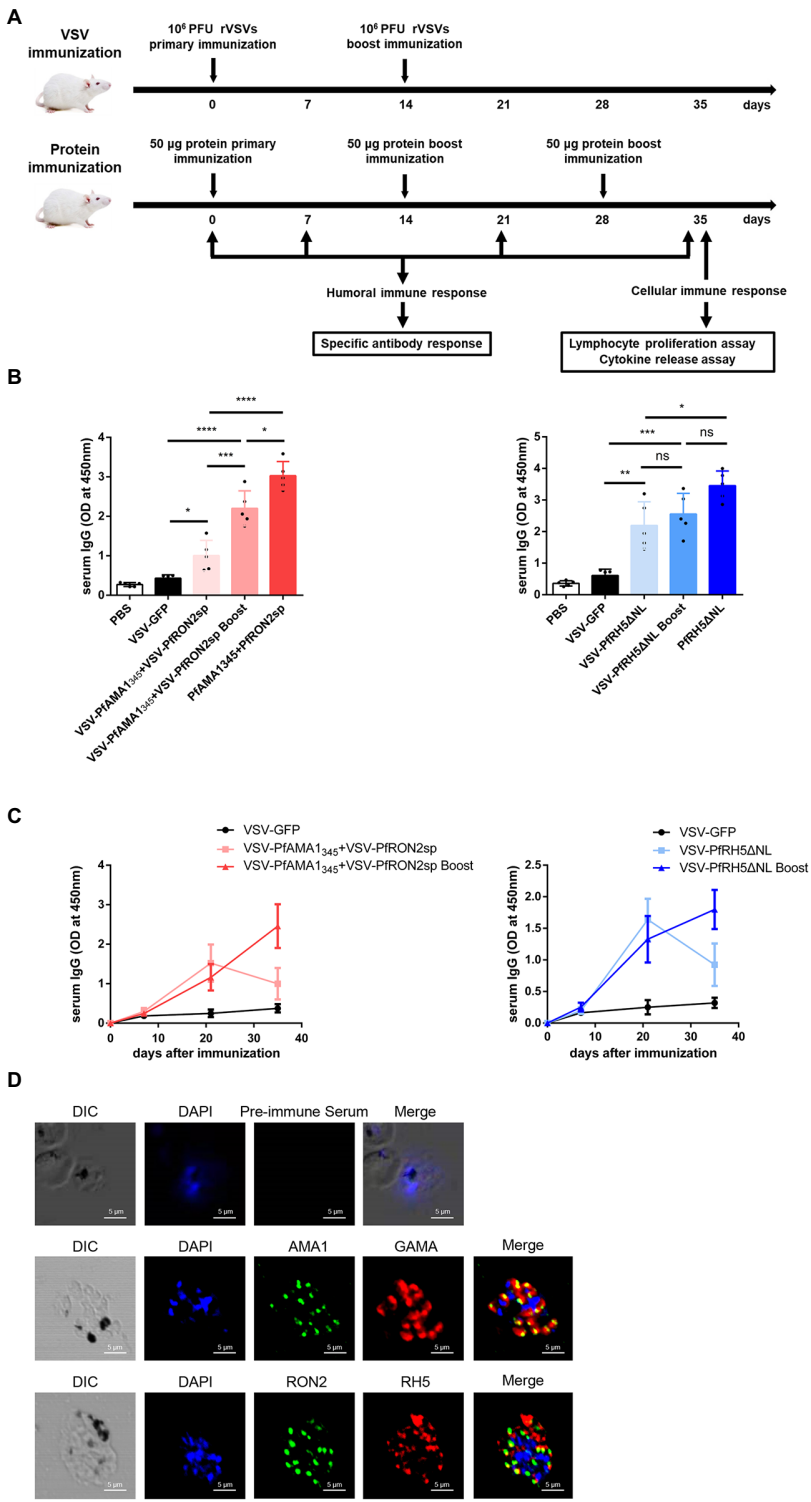
Antigen-specific humoral immune responses induced by rVSVs. **(A)** Schematic representation of animal vaccination and detection strategies. BALB/c mice (n = 5 per group) were intranasally immunized with 25 μl of 10^6^ PFU VSV-PfAMA1_345_ + VSV-PfRON2sp (VSV-PfAMA1_345_ and VSV-PfRON2sp mixture) or VSV-PfRH5ΔNL as single or prime-boost vaccination. The mice were intraperitoneally injected with 50 μg PfAMA1_345_ + PfRON2sp and PfRH5ΔNL antigens three times at the indicated time points. The immune responses were detected at the indicated time points. **(B)** Specific IgG titers were analyzed on day 35 after primary immunization with rVSVs single and prime-boost or proteins by ELISA coated with 5 μg/ml PfAMA1_345_ + PfRON2spor PfRH5ΔNL proteins (peptides). **(C)** The trend of specific IgG antibodies in mice immunized with (Continued)Figure 3 (Continued)VSV-PfAMA1_345_ + VSV-PfRON2sp or mice inoculated with VSV-PfRH5ΔNL on days 0, 7, 21, and 35 after primary immunization. **(D)** Subcellular localization of PfAMA1, PfRH5, and PfRON2 proteins. The parasite was labeled with antisera against PfAMA1_345_, PfRH5ΔNL, and PfRON2sp in the microneme, rhoptry, and rhoptry neck. PfGAMA was used as the marker of PfAMA1. The pre-immune serum was used as the negative control. Nuclei were stained with DAPI and appeared as blue in merged images. The bar represents 5 mm. DIC represents Differential Interference Contrast. One-way AVONA was used to perform statistical analysis. Error bars indicate standard deviation (SD). Ns, no significant, ^*^p < 0.05, ^**^p < 0.01, ^***^p < 0.001 and ^****^ *p* < 0.0001.

For assessing the humoral and cellular immune responses induced by rVSVs, the mice were immunized *via* the intranasal route with 10^6^ PFU rVSVs or boosted with the same dose. The mice were injected with homologous fragments of the three proteins simultaneously *via* the intraperitoneal route, as indicated in the vaccination strategy (Figure 3A). The Pf-specific antibodies were detected in the serum of the mice. The results revealed a significant increase in serum IgG level in mice immunized with a single and booster dose of VSV-PfAMA1_345_ + VSV-PfRON2sp and VSV-PfRH5ΔNL regimens compared to mice immunized with VSV-GFP (Figure 3B). In the mice immunized with the combination of VSV-PfAMA1_345_ and VSV-PfRON2sp, the booster immunization could significantly induce the IgG responses compared to a single dose of vaccination (Figure 3B). Nevertheless, the traditional protein vaccine immunization could still produce a high level of antibody titers (Figure 3B). Furthermore, an increase in antibody levels induced by rVSVs peaked at 3 weeks post-immunization; however, a decrease in antibody levels was observed in the next weeks after a single dose of immunization. Interestingly, an increase in antibody levels was observed 5 weeks after booster immunization (Figure 3C). To determine the specificity of antibodies generated and detect antigens in parasite lysates of *Pf*, immunofluorescence was performed on the serum collected from VSV-*Pf*-immunized mice. The results confirmed that the antisera could recognize the corresponding antigens. PfAMA1 was localized in the microneme, RH5 was localized in rhoptry, and RON2 was localized in the rhoptry neck organelles of the parasite (Figure 3D). These results indicate that strengthening immunization could effectively enhance the production of serum IgG levels and extend the duration of antibody maintenance.

**Figure 4:**
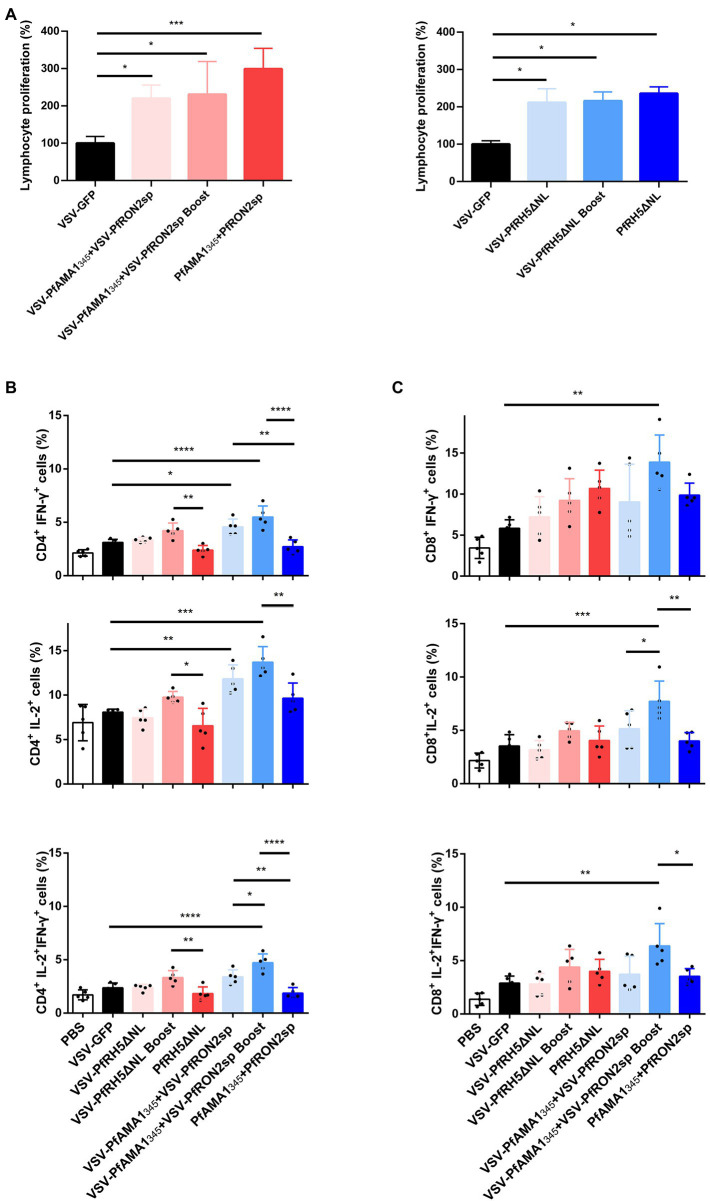
Antigen-specific cellular immune responses induced by immunization with VSV-based vaccines. Spleen cells were collected from immunized mice after 5 weeks of immunization **(A)** Splenic lymphocyte proliferation was assessed using MTT assay after stimulation with 5 μg/ml PfAMA1_345_ + PfRON2sp or PfRH5ΔNL proteins (peptides). VSV-GFP was used as the control. **(B,C)** The frequency of CD4^+^ and CD8^+^ T cells secreting IFN-γ or IL-2 alone was determined by flow cytometry after stimulation with 5 μg/ml PfAMA1_345_ + PfRON2sp mixture or PfRH5ΔNL proteins (peptides) *in vitro* for 24 h. Treatment with PMA (50 ng/ml), ionomycin (1 μg/ml), and bafilomycin A (1 μg/ml) for 6 h. The frequency of CD4^+^ and CD8^+^ T cells secreting IFN-γ and IL-2 were determined by flow cytometry after the same stimulation with proteins (peptides) and treatment with PMA, ionomycin, and bafilomycin A. Error bars indicate SD. One-way AVONA was used to perform statistical analysis.^*^p < 0.05, ^**^p < 0.01, ^***^p < 0.001 and ^****^p < 0.0001.

The proliferation of specific lymphocytes was assessed using the MTT assay to investigate antigen-specific T-cell immune responses induced by rVSV immunization in the spleen. In mice immunized with rVSV single and prime-boost vaccination, a significant increase in the proliferation of specific T cells was observed compared to mice immunized with VSV-GFP (Figure 4A). Additionally, a significant increase in IFN-γ and IL-2-secreting CD4^+^ T and CD8^+^ T cells were observed in mice immunized with VSV-PfAMA1_345_ + VSV-PfRON2sp using prime-boost regimen compared with the VSV-GFP group (Figures 4B,C). An increase in IFN-γ and IL-2 secretion by CD4^+^ T cells was observed in mice immunized with a rVSV boosting regimen, compared to mice immunized with PfAMA1_345_ + PfRON2sp and PfRH5NLΔNL protein only vaccination. Together, these results demonstrate that VSV-based vaccines targeting *P. falciparum* invasion-related antigens could induce antigen-specific T-cell immune responses.

### rVSV immunized mice antisera inhibits *Plasmodium falciparum* invasion *in vitro*

**Figure 5:**
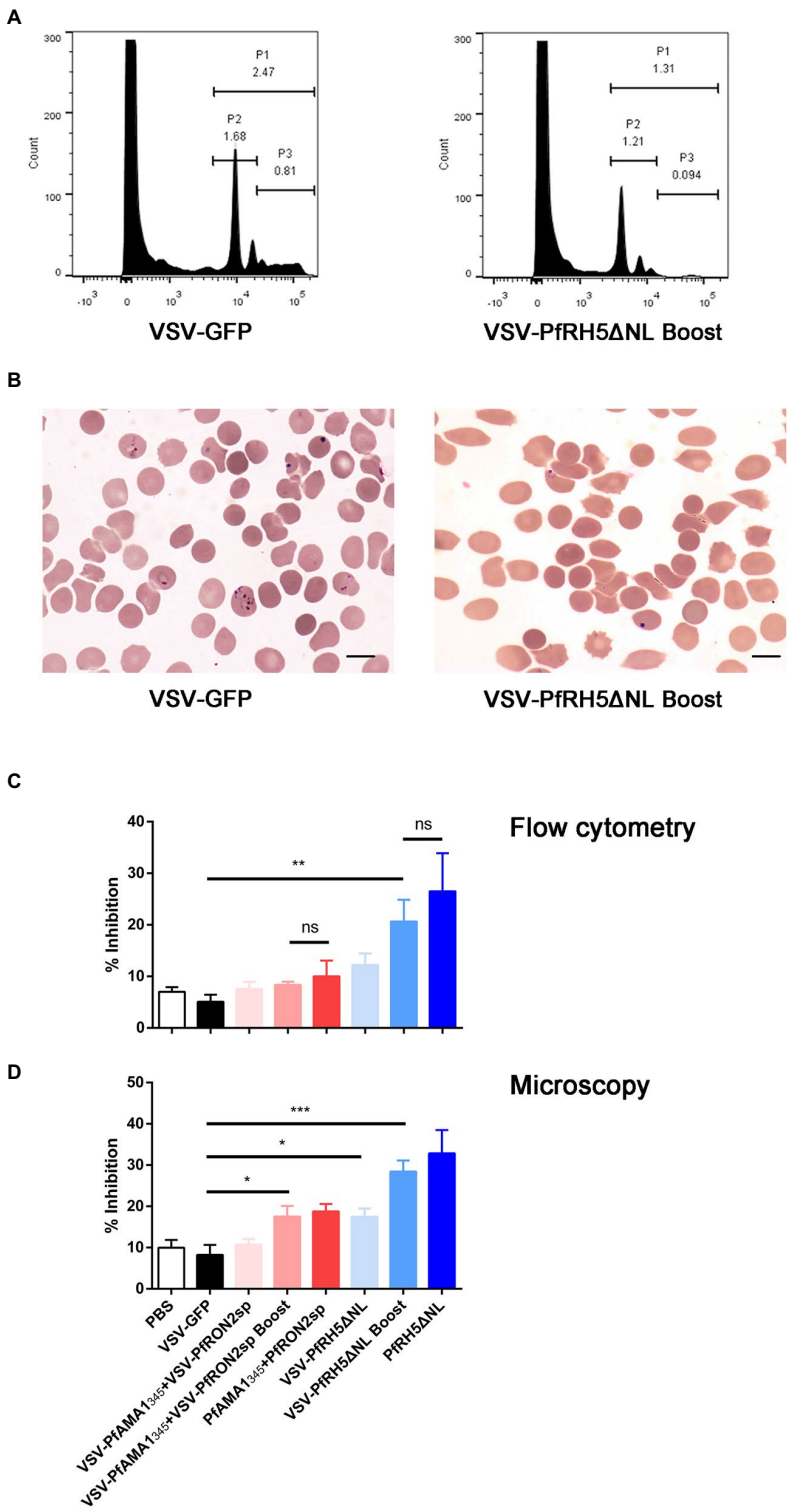
Inhibition efficiency of anti-PfAMA1_345_ + PfRON2sp and anti-PfRH5ΔNL antisera. **(A)** Parasitemia analysis were determined by flow cytometry using SYBR Green staining. P1 represents *Plasmodium falciparum*-infected erythrocytes, P2 represents ring forms after infecting erythrocytes with *P. falciparum*, P3 represents schizonts after infecting erythrocytes with *P. falciparum*. **(B)** Parasitemia analysis using microscopy. The bar represents 10 mm. **(C)** Inhibition of parasite invasion was determined using flow cytometry **(C)** and microscopy **(D)**. Error bars indicate SD. One-way AVONA was used to perform statistical analysis. Ns, no significant, ^*^p < 0.05, ^**^p < 0.01, and ^***^ *p* < 0.001.

The GIA and invasion inhibition assay are widely used functional assays in blood-stage vaccine screening. Therefore, we evaluated the inhibitory effects of antisera from mice immunized with different vaccination strategies on the invasion by the *P. falciparum* 3D7 strain *in vitro*. The microscopic examination and flow cytometry results showed that antisera derived from VSV-PfRH5ΔNL prime-boost immunization regimen could significantly inhibit the parasitic invasion compared to antiserum derived from mice immunized with VSV-GFP (Figures 5A–C). Furthermore, microscopic analysis revealed that VSV-PfRH5NLΔNL single vaccination and VSV-PfAMA1_345_ + VSV-PfRON2sp prime-boost immunization showed a significant inhibitory effect compared to VSV-GFP groups (Figure 5C). In addition, no significant difference in inhibition of invasion by *P. falciparum* 3D7 was observed on treatment with antisera derived from recombinant VSV immunization and homologous protein immunization (Figure 5C). These results indicated that single and prime-boost regimen of VSV-PfRH5ΔNL produced antibodies could inhibit parasite invasion.

### VSV-PyAMA1_343_ and VSV-PyRON2sp vaccination protects against *Plasmodium yoelii* challenge

**Figure 6:**
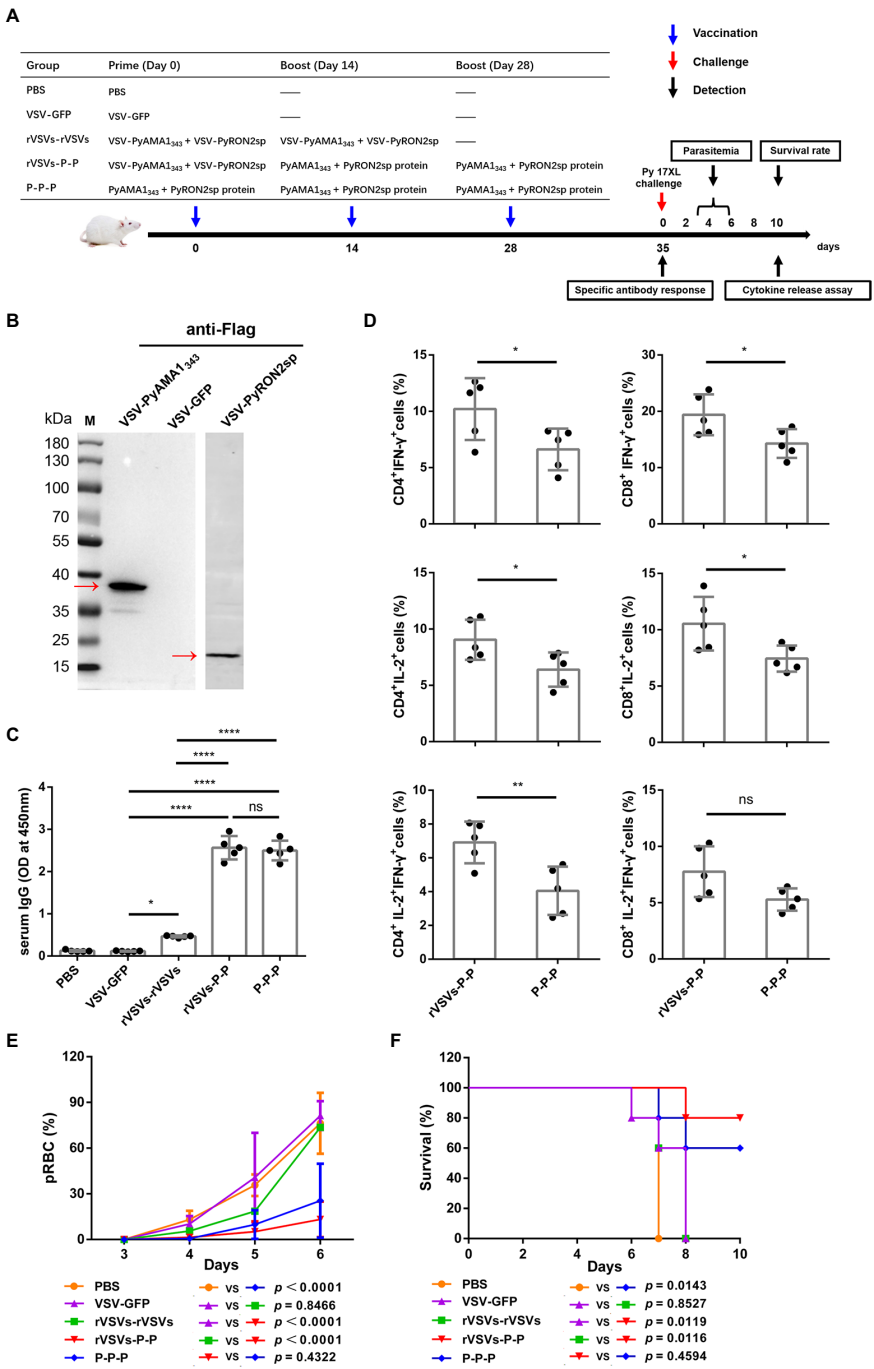
Antigen-specific immune response and immune protection induced by rVSVs. **(A)** The workflow of vaccination, challenge, and detection procedure. BALB/c mice (*n* = 5 per group) were intranasally immunized with 25 μl of 10^6^ PFU VSV-PyAMA1_343_ + VSV-PyRON2sp (VSV-PyAMA1_343_ and VSV-PyRON2sp mixture) through prime-boost vaccination. The mice were intraperitoneally injected with 50 μg PyAMA1_345_ + PyRON2sp antigens three times at the indicated time points. Mice were infected with Py17XL with 5 × 10^5^ perythrocytes 35 days after first immunization. Immune responses were detected at the indicated time points. **(B)** Western blot analysis of expression of PyAMA1_343_ and PyRON2sp in BSR-T7 cells that (Continued)Figure 6 (Continued)infected with recombinant VSV (MOI = 10 for 12 h) using anti-Flag antibodies. **(C)** Specific IgG titers were analyzed on day 35 after primary immunization with rVSVs single and prime-boost or proteins by ELISA coated with 5 μg/ml PyAMA1_343_ + PfRON2sp proteins. One-way AVONA was used **(D)** The frequency of CD4^+^ and CD8^+^ T cells secreting IFN-γ or IL-2 alone was determined by flow cytometry after stimulation with 5 μg/ml PyAMA1_343_ + PyRON2sp mixture proteins *in vitro* for 24 h. Treatment with PMA (50 ng/ml), ionomycin (1 μg/ml), and bafilomycin A (1 μg/ml) for 6 h. The frequency of CD4^+^ and CD8^+^ T cells secreting IFN-γ and IL-2 were determined by flow cytometry after the same stimulation with proteins and treatment with PMA, ionomycin, and bafilomycin A. One-way AVONA was used to test the differences. **(E)** Analysis of the infection rate of RBC *via* blood smears on days 3, 4, 5, and 6 post-challenge with Py17XL. Two-way AVONA was used to test the differences. **(F)** Survival rates of different immunization strategies in inoculated mice post-challenge. Log-rank (Mantel-Cox) tests were used to analyze mouse survival curves. Error bars indicate SD. Ns, no significant, ^*^p < 0.05, ^**^p < 0.01, ^***^p < 0.001, and ^****^p < 0.0001.

Primates are the only host of *P. falciparum*, and conducting *in vivo* experiments in primates could be challenging (Beignon et al., 2014). Therefore, *P. yoelii,* which can infect rodents and cause malaria, was used as an alternative model to evaluate the efficacy of VSV-based vaccines (Figure 6A). RH5 has no homologous protein in *P. yoelii*; therefore, we could not construct recombinant VSV for *in vivo* experiments. To investigate the protective effect of VSV-based malaria vaccines, a recombinant VSV vaccine was constructed expressing AMA1_343_ and RON2sp of *P. yoelii*, which are homologous fragments of PfAMA1_345_ and PfRON2sp. The cytopathic effect on BSR-T7 cells was observed under a microscope, indicating that VSV-PyAMA1_343_ and VSV-PyRON2sp were successfully packaged (Supplementary Figure S2B). Furthermore, PyAMA1_343_ and PyRON2sp were expressed in recombinant VSVs and verified by western blotting (Figure 6B). These results confirm that recombinants VSV-PyAMA1_343_ and VSV-PyRON2sp were successfully generated.

Specific IgGs in serum and cytokine secretion by T cells derived from the spleen were investigated to evaluate the efficacy of VSV-PyAMA1_343_ and VSV-PyRON2sp hybrid immunization strategies. The results showed that immunization with rVSVs-P-P and P-P-P induce higher IgG levels in mice after 5 weeks of vaccination compared to mice immunized with rVSVs-rVSVs (Figure 6C). However, no significant difference between the IgG induced by in sera of mice immunized with rVSVs-P-P and P-P-P (Figure 6C). Furthermore, we evaluated if rVSVs-P-P and P-P-P could induce specific cellular immune responses on *P. yoelii* challenge. The results showed that immunization with rVSV-P-P could induce CD4^+^ and CD8^+^ T cells to secrete high levels of IFN-γ and IL-2 in mice compared to immunization with P-P-P (Figure 6D).

BALB/c mice from all the groups were infected with Py17XL by administering 5 × 10^5^ perythrocytes intraperitoneally after 35 days of initial immunization. This will allow us to evaluate whether immunization with rVSVs expressing PyAMA1_343_ and PyRON2sp could protect the mice against *P. yoelii* infection. The microscopic examination revealed that the percentage of perythrocytes was significantly lower in mice immunized with rVSVs-P-P and P-P-P compared to mice immunized with VSV-GFP and PBS, respectively (Figure 6E). On day 6 after the Py17XL challenge, the parasitemia was less than 30% in mice immunized with rVSVs-P-P and P-P-P regimens (Figure 6E). Moreover, all mock-immunized mice died within 8 days of *P. yoelii* challenge. The survival rate of rVSVs-P-P and P-P-P-immunized mice was significantly higher compared to mice immunized with VSV-GFP and PBS, respectively (Figure 6F). Nevertheless, no significant differences in survival rate were observed between mice immunized with rVSVs-P-P and P-P-P (Figure 6F). These results indicate that the rVSVs-P-P and P-P-P immunization strategies protected mice against *P. yoelii* infection and the rVSV prime-protein boost immunization induced stronger polyfunctional T cell responses.

## Discussion

An effective vaccine that successfully eliminates malaria could aid in improving public health. RTS,S/AS01 is the only licensed malaria vaccine undergoing phase III clinical trial. However, the vaccine has moderate efficacy and may not be able to eradicate malaria (Neafsey et al., 2015; Olotu et al., 2016; Tinto et al., 2019; Arora et al., 2021). Despite challenges associated with vaccine development, such as the genetic variation and antigenic diversity of parasites (Neafsey et al., 2021), some strategies, including novel viral vectors as an antigenic delivery platform, have been used to enhance the protective efficacy of the malarial vaccines (Frimpong et al., 2018; Humphreys and Sebastian, 2018). Further, vaccines with viral vector backbone are currently being used against Ebola virus disease in clinical settings (Choi et al., 2021). However, VSV has not been used for malaria vaccine development until now. In this study, we have constructed three recombinant VSV-based vaccines expressing *P. falciparum* gene fragments like AMA1_345_, RON2sp, and RH5ΔNL. Our results show that these vaccines could induce high IgG levels and a strong antigen-specific T-cell immune response in immunized mice. Although single immunization with rVSVs could not induce a strong T cell response, the prime-booster rVSVs regimens inhibited invasion of *P. falciparum in vitro*. Interestingly, the protective efficacy of rVSVs prime-protein boost vaccination was comparable to protein immunization in mice against *P. yoelii* infection. Our results provide novel insights into the development of VSV-based vaccines for malaria.

Various studies have used viral vectors for malaria vaccine development (Ewer et al., 2015), including Modified Vaccinia Virus Ankara (MVA), chimpanzee adenovirus 63 (ChAd63) (Kim et al., 2020), Human Adenovirus Serotype 5 (AdHu5), adeno-associated virus serotype 1 (AAV1) (Yusuf et al., 2019), and AAV8 (Shahnaij et al., 2021). Furthermore, the ChAd63-MVA vaccine encoding multiple epitope string thrombospondin-related adhesion proteins (ChAd63-MVA ME-TRAP) is currently undergoing clinical trials (Kimani et al., 2014; Hodgson et al., 2015; Tiono et al., 2018). However, the protective efficacy of the vaccine is still unsatisfactory in infants in the high malaria-endemic region (Tiono et al., 2018). ChAd63 and MVA viral vector vaccines encoding Pfs25-IMX313 induce T-cell and B-cell responses in humans; nevertheless, the efficacy of antibodies in serum to reduce transmission is weak (de Graaf et al., 2021). To the best of our knowledge, our study is the first to use the rVSV vector to develop a malaria vaccine. Further, our results show that booster immunization with VSV-PfRH5ΔNL and VSV-PfAMA1_345_ + VSV-PfRON2sp could trigger high IgG levels in serum and promote the proliferation of specific lymphocytes (Figures 3B, 4A). Moreover, the vaccine can significantly inhibit erythrocyte invasion (Figure 5C), thereby indicating that the rVSV vaccines targeting *Plasmodium* blood-stage antigens could induce functional immune responses.

The humoral immune response plays an important role in conferring protection and developing immunity against parasitic natural infections and vaccine inoculation (Boyle et al., 2017; Gonzales et al., 2020). Our results are consistent with the previous studies, which used VSV as a vector for vaccine development, such as vaccines against COVID-19 (Case et al., 2020) and the Marburg virus (Marzi et al., 2018). These studies have demonstrated that immunizing mice with rVSVs expressing microbial antigens could induce high antibody titers. A consistent increase in IgG levels was observed in sera of mice immunized with prime-boost regimens such as VSV-PfRH5ΔNL or VSV-PfAMA1_345_ + VSV-PfRON2sp (Figure 3B). However, the antibody titers began to decline after 3 weeks of a single dose of immunization (Figure 3C). To address the concerns regarding the loss of potency to generate rVSV-induced functional antibodies after a single immunization, we have developed a new rVSV-P–P strategy. This strategy could induce higher antibody levels compared to the rVSVs-rVSVs regimen; in fact, this strategy can induce antibody levels comparable to those produced by the protein vaccine (Figure 6C).

T cells are indispensable for attenuating the replication of the *Plasmodium* parasite and reducing the severity of malaria (Kurup et al., 2019). Furthermore, IFN-γ plays a critical role in the immune response against *Plasmodium* by inducing phagocytosis and activating macrophages to promote the killing of parasites and other phagocytic cells (Gbedande et al., 2020). Moreover, IFN-γ produced by CD4^+^T cells in response to *Plasmodium* antigen is IL-2 dependent (Kimura et al., 2010). A subunit malaria vaccine confers protection against sporozoite challenge by increasing the production of IL-2-secreting CD4^+^T cells (Chawla et al., 2019). Our results showed that single and booster immunization with VSV-PfAMA1_345_ + VSV-PfRON2sp significantly increases the levels of IFN-γ-and IL-2-secreting CD4^+^T cells compared to VSV-GFP immunization (Figure 4B). IFN-γ activates phagocytosis and killing of parasites, whereas parasitic invasion is primarily dependent on ligand-receptor interactions between parasites and erythrocytes (Patarroyo et al., 2020). Although rVSV immunization could increase the secretion of cytokines, no significant differences in inhibiting parasitic invasion were observed between rVSV regimens and homologous protein immunization (Figure 5C). Studies have shown that MHC-I aids in recognition of *P. vivax* and *P. yoelii*-infected reticulocytes by cytotoxic CD8^+^T cells. Further, cytotoxic T-lymphocytes aid in parasite clearance during the blood stage of malaria (Junqueira et al., 2018; Hojo-Souza et al., 2020). Our results show a significant increase in levels of IFN-γ and IL-2-secreting CD8^+^ T cells in the spleen of mice immunized with VSV-PfAMA1_345_ + VSV-PfRON2sp prime-boost regimen (Figure 4C). This indicates that the VSV based vaccines could effectively induce CD8^+^ T cell-mediated immune response. A clinical study reported the involvement of cytotoxic CD8^+^ T cells in the pathogenesis of malarial complications in humans (Kaminski et al., 2019); however, the role and underlying mechanisms of CD8^+^ T cell responses in *Pf* blood-stage vaccine are unclear and should be explored further.

Although antigen and viral vector-based vaccines can induce CD4^+^ and CD8^+^T cell responses, T-cell responses induced by vaccines are influenced by multiple factors, including the type of viral vectors and antigens of specific pathogens (Sasso et al., 2020). For example, non-human primate adenoviruses (NHPAd) are vaccine vectors that can induce a high level of CD4^+^ and CD8^+^T cell responses (Sasso et al., 2020). Whereas the proportion of IFN-γ and IL-2 double-positive CD8^+^T cells induced by the viral antigen coxsackievirus B3(CVB3)-VP1-VSV was less compared to CD4^+^ T cells (Wu et al., 2014). Our results showed that rVSV vaccines containing malaria antigens could induce both CD4^+^ and CD8^+^ T cells secreting IFN-γ and IL-2 (Figures 4B,C). Malaria vaccines with virus-like particles (Lee et al., 2020) and AAV8 (Shahnaij et al., 2021) as vectors can induce the production of high levels of memory T cells, which is an important defense mechanism against malaria. Whether rVSV vaccines could also induce the production of memory T cells should be investigated further (Krzych et al., 2014; Moncunill et al., 2017). Regarding immunization routes, the intranasal route is commonly used for administering VSV-based vaccines, such as STAR-CoV2 (Case et al., 2020). Intranasal inoculation of VSV vaccines could induce mucosal immunity in addition to humoral and cellular immunity (Wu et al., 2014). A recent study has shown that sporozoites inoculated in the skin induce circulating IgA production, and IgA monoclonal antibody reduces the parasitemia in the liver of mice (Tan et al., 2021). Whether the rVSV malaria vaccine induces mucosal immunity should be further investigated.

The microbial infection model could be used to evaluate the effectiveness of vaccines and investigate the underlying mechanisms of protective immunity *in vivo*. Rodent parasites, including *P. yoelii*, have been widely used to study parasite biology and mammalian immune responses to malaria (De Niz and Heussler, 2018). To verify the effects of recombinant VSV vaccines *in vivo*, we explored the protective efficacy of different vaccine immunization strategies using Py17XL as a model. After the Py17XL challenge, a significant decrease in the parasitemia of mice immunized with rVSVs-P-P and P-P-P groups was observed (Figure 6E). A significant increase in the survival rate of the mice immunized with rVSVs-P-P and P-P-P groups was observed (Figure 6F). Further, a significant increase in IFN-γ and IL-2 secretion by T cells was observed in mice immunized with rVSVs-P-P regimen compared to the mice immunized with P-P-P (Figure 6D). Together, these results indicate that VSV-prime and protein-boost regimen primarily induced high immunogenicity.

Notably, the rVSVs-rVSVs immunization strategy conferred weak protection, likely due to the complex immune mechanisms developed by protozoans. Further, the factors contributing to the reduced efficacy of homologous rVSV regimens include the presence of potential anti-vector antibodies. The pre-existing antibodies against VSV could probably reduce the efficacy of the homologous rVSV regimens. This could inhibit the replication of VSV vectors (Poetsch et al., 2019) and may impair the protective immunity against malaria. In addition, a previous study has suggested that the protective efficacy of the VSV-Ebola vaccine was dependent on antigen dose and immunization route (Jones et al., 2007). Thus, investigating more candidate antigens and different routes of immunization for using VSV vaccines is necessary.

In conclusion, we established a novel VSV-based vaccine approach for malaria and evaluated the vaccine efficacy. Our results demonstrated that rVSVs expressing AMA1_345_, RON2sp, and RH5ΔNL gene fragments of *P. falciparum* could effectively induce specific humoral and cellular immune responses as well as inhibit *P. falciparum* invasion. Furthermore, the rVSVs-P-P immunization strategy was more efficacy in protection against Py17XL infection. Our study demonstrated the use of VSV as a vaccine vector and provided new insights into effective malaria prevention.

## Data availability statement

The raw data supporting the conclusions of this article will be made available by the authors, without undue reservation.

## Ethics statement

The animal study was reviewed and approved by Animal Ethics Committee of Jiangnan University [JN. No. 20180615t0900930 (100)].

## Author contributions

YC and CD conceived this study. YS and XS designed the research protocol, wrote the manuscript, and performed the data acquisition and analysis. XS, YS, YY, and HF performed laboratory work. YS, XS, YC, CJ, FL, and ETH conceived the data handling and analysis process and reviewed the manuscript. JX, ETH, XH, and YC interpreted the results and assisted in writing the manuscript. All authors read and approved the final version of the manuscript.

## Funding

This study was funded by grants from the National Natural Science Foundation of China (No. 81871681), the Youth project of Wuxi Health Commission (No. Q202124) and Wuxi Medical Key Discipline (No. ZDXK2021002).

## Conflict of interest

The authors declare that the research was conducted in the absence of any commercial or financial relationships that could be construed as a potential conflict of interest.

## Publisher’s note

All claims expressed in this article are solely those of the authors and do not necessarily represent those of their affiliated organizations, or those of the publisher, the editors and the reviewers. Any product that may be evaluated in this article, or claim that may be made by its manufacturer, is not guaranteed or endorsed by the publisher.

## Supplementary material

Supplementary Figure S1 **(A)** PfRON2sp peptide was purified by chromatography and identified by mass spectrometry. The lower panel showed the peak time, area under the peak and protein concentration of the peak during PfRON2sp peptides were harvest.

Supplementary Figure S2 **(A)** Microscopic images of BSR-T7 cells infected with VSV-PfAMA1_345_, VSV-PfRH5ΔNL and VSV-PfRON2sp 24 h post-infection. Non-infected cells were used as mock control, and VSV-GFP-infected cells were used as positive control. The bar represents 20 mm. **(B)** Images of the microscope for cytopathic effect of BSR-T7 cells infected with VSV-PyAMA1_343_ and VSV-PyRON2sp 24 h post-infection. Non-infected cells as mock control, and VSV-GFP-infected cells as positive control. The bar represents 20 mm.
