# Supplementary figures and images for: Vesicular stomatitis virus-based vaccine targeting plasmodium blood-stage antigens elicits immune response and protects against malaria with protein booster strategy
Source: Front Microbiol. 2022 Nov 24;13:1042414. doi: 10.3389/fmicb.2022.1042414 (PMC9731671; doi:10.3389/fmicb.2022.1042414)

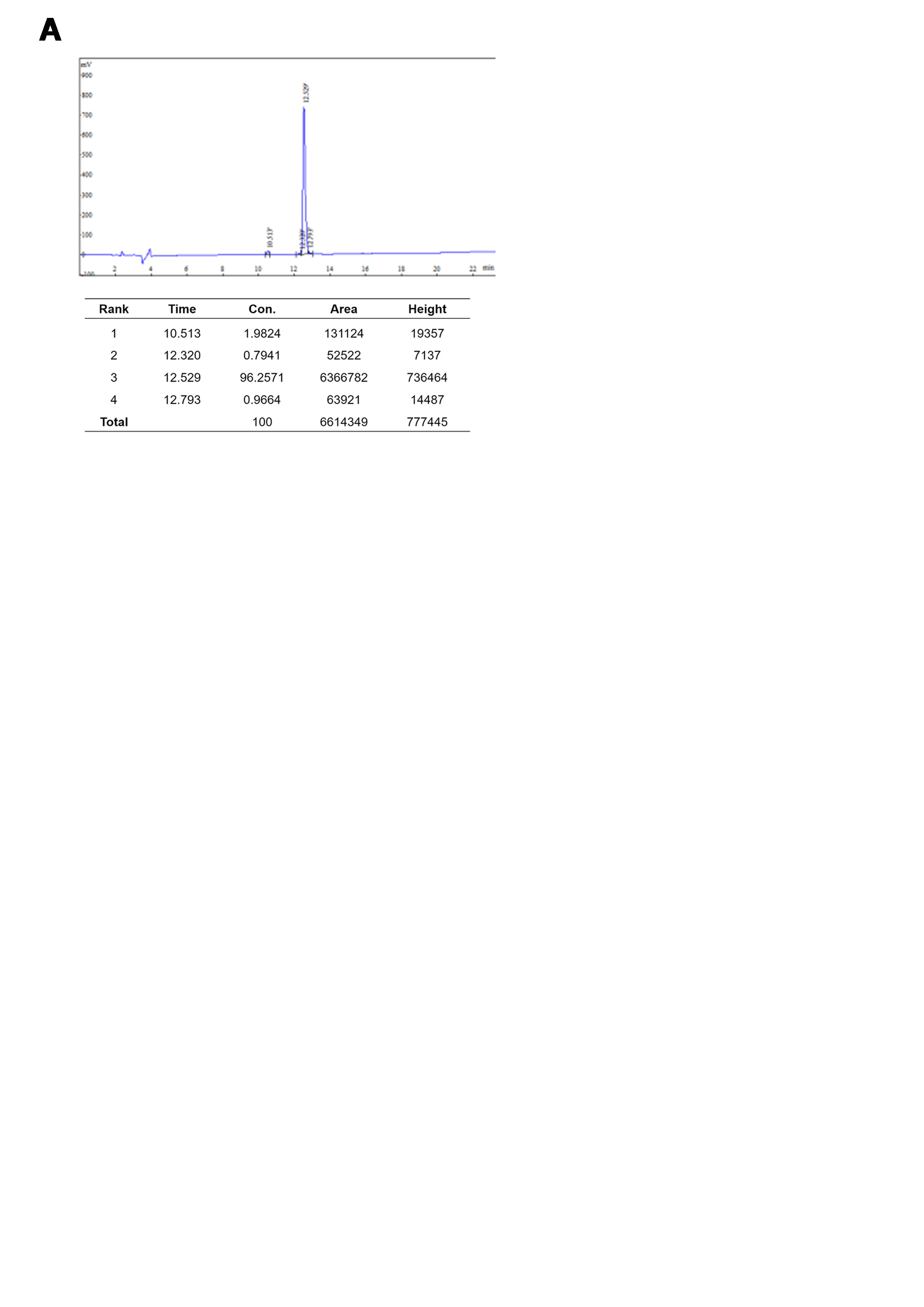

Supplement: Supplementary Figure S1 — (A) PfRON2sp peptide was purified by chromatography and identified by mass spectrometry. The lower panel showed the peak time, area under the peak and protein concentration of the peak during PfRON2sp peptides were harvest. [file Image_1.TIF]

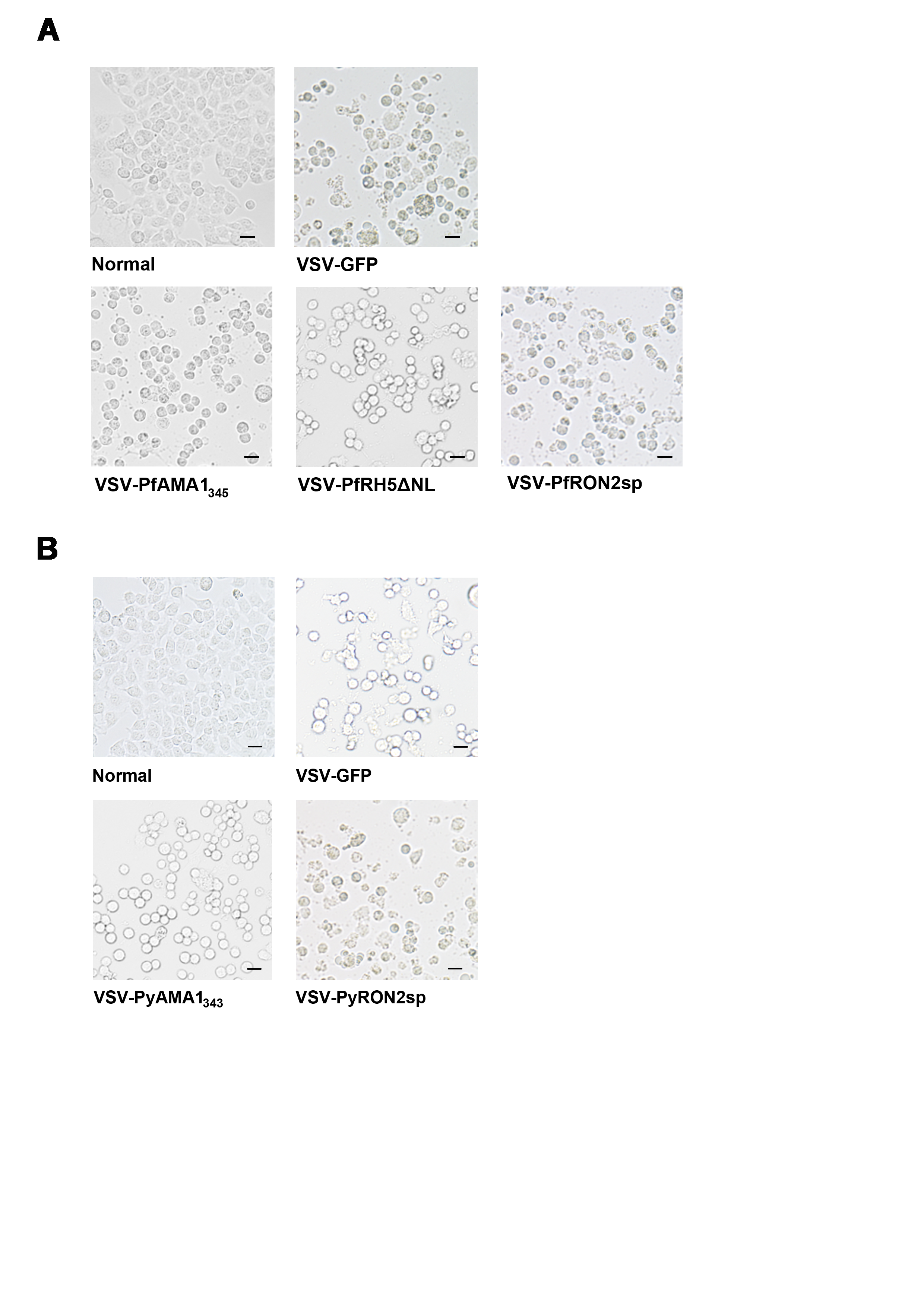

Supplement: Supplementary Figure S2 — (A) Microscopic images of BSR-T7 cells infected with VSV-PfAMA1345, VSV-PfRH5ΔNL and VSV-PfRON2sp 24 h post-infection. Non-infected cells were used as mock control, and VSV-GFP-infected cells were used as positive control. The bar represents 20 mm. (B) Images of the microscope for cytopathic effect of BSR-T7 cells infected with VSV-PyAMA1343 and VSV-PyRON2sp 24 h post-infection. Non-infected cells as mock control, and VSV-GFP-infected cells as positive control. The bar represents 20 mm. [file Image_2.TIF]
